# Scoping Review of Neuroimaging Studies Investigating Frailty and Frailty Components

**DOI:** 10.3389/fmed.2018.00284

**Published:** 2018-10-08

**Authors:** David López-Sanz, Isabel Suárez-Méndez, Raquel Bernabé, Natalia Pasquín, Leocadio Rodríguez-Mañas, Fernando Maestú, Stefan Walter

**Affiliations:** ^1^Laboratory of Cognitive and Computational Neuroscience (UCM-UPM), Centre for Biomedical Technology (CTB), Technical University of Madrid (UPM), Madrid, Spain; ^2^Department of Experimental Psychology, Complutense University of Madrid (UCM), Madrid, Spain; ^3^Fundación Para la Investigación Biomédica, Getafe University Hospital, Madrid, Spain; ^4^Geriatrics Department, Getafe University Hospital, Madrid, Spain; ^5^Centro de Investigación Biomédica en Red Fragilidad y Envejecimiento Saludable (CIBERFES), Madrid, Spain; ^6^Centro de Investigación Biomédica en Red en Bioingeniería, Biomateriales y Nanomedicina (CIBER-BBN), Zaragoza, Spain; ^7^Department of Epidemiology and Biostatistics, University of California, San Francisco, San Francisco, CA, United States

**Keywords:** frailty, neuroimaging (anatomic and functional), review, gait speed, grip strength

## Abstract

**Background:** Neuroimaging techniques are a cornerstone for diagnosing and investigating cognitive decline and dementia in the elderly. In frailty research, the physical as opposed to the cognitive domain of the aging process, neuroimaging studies are less common. Here we systematically review the use of neuroimaging techniques in frailty research.

**Methods:** We searched PUBMED for any publication reporting the association between neuroimaging markers and frailty, following Fried's original definition, as well as its determining phenotypes: gait speed, grip strength, fatigue and recent weight loss in the non-diseased population older than 65 years.

**Results:** The search returned a total of 979 abstracts which were independently screened by 3 reviewers. In total, 17 studies met the inclusion criteria. Of these, 12 studies evaluated gait speed, 2 grip strength, and 3 frailty (2 Fried Frailty, 1 Frailty Index). An association between increased burden of white matter lesions, lower fractional anisotropy, and higher diffusivity has been associated consistently to frailty and worse performance in the different frailty components.

**Conclusions:** White matter lesions were significantly associated to frailty and frailty components thus highlighting the potential utility of neuroimaging in unraveling the underlying mechanisms of this state. However, considering small sample size and design effects, it is not possible to completely rule out reverse causality between frailty and neuroimaging findings. More studies are needed to clarify this important clinical question.

## Introduction

The number of older people in the global population is rapidly growing. From 2013 to 2060 the percentage of the population aged over 65 years is projected to increase from 18 to 28% and the proportion of those aged over 80 years will rise from 5 to 12% (1). Increased longevity raises social and economic challenges and has deep implications for the planning and delivery of healthcare. Indeed, as the number of older people rises, so does the number of people with age-related disability and dependence that require support with daily activities, healthcare services and/or institutionalization.

The transition from a robust status to one of age-related disability is usually preceded by a physiological state termed frailty (2, 3). Although frailty can be characterized using classical clinical phenotypes and laboratory-based biomarkers, a universally accepted definition of frailty remains to be agreed upon (4, 5). The most widely accepted definition of frailty is “an age-associated biological syndrome, characterized by a decrease of the biological reserve and resistance to stress due to a decline in several physiological systems. This places the individual in a special risk category when facing minor stressors and is associated with poor outcomes (disability, hospitalization and death)” (6). The most prominent approach used to assess frailty is using Fried′s Frailty Criteria (7). Following this model, frailty is diagnosed based on the presence of at least three of the five physical attributes and capabilities of an individual. These include: weight loss (unintentional weight loss of 4.5 kg or more in the last year), exhaustion (self-reported), physical inactivity, slow walking speed, and weakness (low grip strength).

Many research initiatives, including the large scale European FRAILOMIC initiative, investigate OMIC factors associated to frailty (4, 8). In a recent seminar published in the Lancet (6), the authors discuss under the subheading ≪The Frail Brain≫ only the structural and physiological changes taking place in the brain that are known to be associated with chronological age but not with frailty specifically. They reference the relationship between frailty and cognition as an example of the frail brain rather than answering which specific structural and physiological changes in the brain are associated with frailty.

In this scoping review the objective is to summarize the use of neuroimaging techniques in investigating Fried Frailty in the non-diseased, elderly population. In addition, we want to narratively outline whether current knowledge supports an overlap with dementia research.

## Methods

Following PRISMA methodology, for this scoping review we searched PubMed looking for works published prior to February 2018 (9–11).

We used the following query:

Neuroimaging [MESH] AND (Frailty OR (gait velocity OR gait speed) OR (grip strength OR muscle strength) OR fatigue OR weight loss)

**Figure 1:**
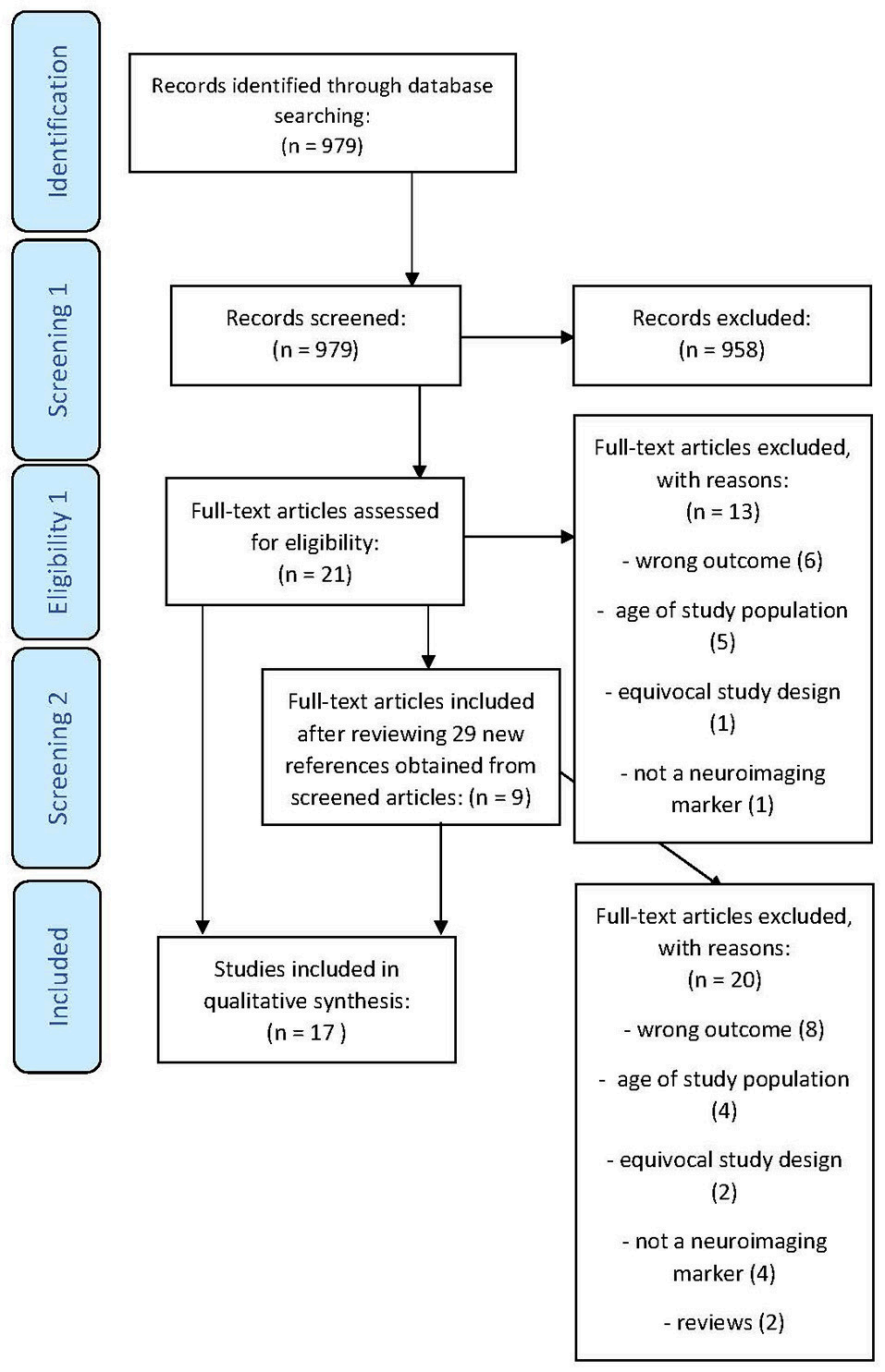
PRISMA flow diagram.

We restricted the result set to those investigating humans using the PubMed filter functionality and adults older than 65 years. Nine Hundred and Seventy-Nine abstracts were reviewed independently by three researchers (SW, RB, and NP) with the help of abstrackr software without using the prediction algorithm (12). We excluded 958 papers, including those that investigated the relationship between neuroimaging markers and frailty parameters such as gait speed or grip strength only in diseased populations (e.g., Parkinson′s Disease, Stroke, etc.) after reviewing the abstract. Of the remaining 21 papers that passed through full text screening, 13 were excluded for different reasons: not investigating frailty or its components (*n* = 6), study design not restricted to a population of 65 years of age or older (*n* = 5), inadequate study design (*n* = 1) and not including a neuroimaging marker (*n* = 1). When reviewing the references from the 21 articles originally deemed eligible after abstract screening, 9 studies were further considered eligible (Figure 1).

## Results

Of the 17 studies that fulfilled the inclusion criteria (Table 1), 3 studied frailty (2 Fried Frailty, 1 Frailty Index), 2 studies investigated grip strength, and 12 studies investigated gait speed or gait parameters. All but three studies were cross-sectional in nature. Table 2 lists the details of the outcome assessment, the imaging risk factors studied, the application of confounder control, and the conclusions for each of the studies included in this review.

**Table 1:** Descriptive overview of reviewed studies.

**References**	**Year**	N (% female)^*^	**Age (mean, SD)**	**Design**	**Outcome**	**Imaging technique**
**FRAILTY**						
(13)	2017	176 (40.0%)	75.0 (5.2)	Cross-sectional	Frailty	Structural (T1-weighted MRI incl. DTI)
(14)	2014	87 (62.1%)	Median 78 (IQR 74-83)		Frailty	Structural (T2-weighted MRI)
(15)	2001	4735 (42.8%)	72.7 (4.33)	Cross-sectional	Frailty	Structural MRI (Image weighting not specified)
**GRIP STRENGTH**						
(16)	2015	191 (53.4%)	70.3 (4.8)	Cross-sectional	Grip Strength	Functional MRI (Resting State)
(17)	2016	165 (51%)	70.15 (4.50)	Cross-sectional	Grip Strength	Structural (T1-weighted MRI incl. DTI) and Functional (Resting State)
**GAIT SPEED**						
(18)	2010	148 (56.1%)	79 (IQR 76 - 83)	Cross-sectional	Gait Speed	Structural (T1-weighted MRI)
(19)	2012	214 (64.5%)	72.82 (3.77)	Cross-sectional	Gait Speed	Structural (T1-weighted MRI)
(20)	2010	795 (58.9%)	75.6 (5.5)	Cross-sectional	Gait Speed	Structural (T1-weighted MRI)
(21)	2016	265 (57%)	82.9 (2.7)	Cross-sectional	Gait Speed	Structural (T2-weighted MRI incl. DTI)
(22)	2015	30 (55.17%)	72.5 (5.22)	Cross-sectional	Gait Speed	Functional MRI (Resting State)
(23)	2008	104 (61.5%)	85.1 (5.6)	Longitudinal	Gait Speed	Structural MRI (T1 and T2-weighted)
(24)	2009	1702 (60.6%)	72.4 (4.1)	Longitudinal	Gait Speed	Structural MRI (T1 and T2-weighted)
(25)	2007	327 (56.5%)	78.2 (3.9)	Cross-sectional	Gait Speed	Structural (T1 and T2-weighted MRI)
(26)	2005	2450 (57%)	74.4 (4.7)	Longitudinal	Gait Speed	Structural (T1 and T2-weighted MRI)
(27)	1999	50 (62%)	85.1 (7.2)	Cross-sectional	Gait Speed	Structural (T1-weighted MRI)
(28)	2003	97 (40.2%)	78–79	Cross-sectional	Gait Speed	Structural (T2-weighted MRI)
(29)	2000	390 (0%)	72.37 (2.96)	Cross-sectional	Gait Speed	Structural MRI (Image weighting not specified)

* *The n refers to the source population for which the descriptive statistics % female and mean age are reported*.

**Table 2:** Outcome, Imaging Risk Factors, and Conclusions from reviewed studies.

**References**	**Year**	**Study acronym or name (location)**	**Outcome assessment**	**Imaging risk factor**	**Confounder control**	**Conclusion**
**FRAILTY**						
(13)	2017	AMImage	Fried Frailty	White Matter Hyperintensities and Integrity: Fractional Anisotropy (FA), Axial Diffusivity (AD), Radial Diffusivity (RD), and Mean Diffusivity (MD).	Yes	Frail people have higher white matter hyperintensity volume and loss of white matter integrity.
(14)	2014	Seoul National University	Frailty Index estimated from: Score in daily activies, cognitive function, physical performance and serum albumin test.	White Matter Lesions (WML).	Yes	Higher frailty score in those subjects with more WML, thus they conclude both variables to be associated.
(15)	2001	CHS	Fried frailty.	WML, Infarct-like Lesions, Sulcal Prominence and Ventricular Size.	Yes	Frail subjects showed more infarct lesions, increased white matter abnormalities and increased ventricular size, no effect on sulcus size was found.
**GRIP STRENGTH**						
(16)	2015	LHAB	Grip Strength with hydraulic hand dynamometer.	Functional Connectivity between Left Motor Cortex, Left Putamen, Right Lobule V, R Lobule VIII.	No	Sensorimotor cortex connectivity is positively associated with grip strength.
(17)	2016	LHAB	Grip Strength with hydraulic hand dynamometer.	White Matter Integrity: FA, MD, RD, AD in Cingular Bundle; approximated Default Mode Network Connectivity	Yes	RD was significantly associated to grip strength, resting state functional connectivity was not.
**GAIT SPEED**						
(18)	2010	MCSA	Gait Speed using a 4.88 m digitized walkway system.	White Matter Hyperintensities.	No	Higher white matter intensity volumes across all regions were associated to lower gait speed.
(19)	2012	CHS	Gait Speed using a 4.57 m course and the average of 2 measurements.	Gray Matter Volume of the Prefrontal Area.	Yes	Smaller prefrontal area gray matter volume is associated with slower gait speed.
(20)	2010	AGES	Gait Speed using a 6 m course and the average of 2 measurements.	Magnetization Transfer Ratio, White Matter Hyperintensities, Brain Athrophy and Brain Infarcts.	Yes	Lower magnetization transfer ratio, higher white matter intensity volume and generalized brain atrophy but not brain infarcts were associated to slower gait speed.
(21)	2016	HealthABC	Gait Speed using an 8 m computerized walkway.	White Matter Hyperintensities and FA.	Yes	Higher white matter lesion volume was associated with slower gait speed, a significant interaction was observed between white matter hyperintensities and FA. In high FA individuals, the association was non-significant.
(22)	2015	CCMA	Gait Speed on 6.10 m computerized walkway.	rs-FMRI and ICA Decomposition.	No	Gait Speed associated with well-established sensorimotor, visual, vestibular, and left fronto-parietal resting-state networks in older adults.
(23)	2008	Oregon Brain Aging Study	Gait Speed using a 9 m course.	Periventricular, Subcortical and Total WMH, Total Brain Volume, Hippocampal Volume, CSF Volume.	Yes	Higher baseline total and periventricular white matter hyperintensities was related to more pronounced change in gait speed and number of steps during follow-up. Higher rate of periventricular white matter hyperintensities accumulation was associated with increased gait slowing.
(24)	2009	3C study France	Gait Speed using a 6 m course.	White Matter Lesions.	Yes	Periventricular WML volume was associated with slow gait speed in those subjects above 90th percentile of WML volume, deep WML volume was not. Baseline total WML volume predicted walking speed decline in follow-up.
(25)	2007	CHS	Gait Speed using a 4.57 m course and Balance checking the ability to hold semitandem position for at least 10 s.	Gray Matter Volume of ROIs known to be associated with mobility.	Yes	Smaller gray matter volumes remained associated with slow gait and poor balance after cofounder control in LH smaller cerebellum and dorsolateral prefrontal regions (slower gait) and RH basal ganglia, superior posterior parietal cortex and cerebellum (balance difficulty).
(26)	2005	CHS	Gait Speed using a 4.57 m course.	Ventricular Enlargement, White Matter Hyperintensities, Subcortical and Basal Ganglia Small Brain Infarcts.	Yes	Presence of structural brain abnormalities was associated with greater risk of incident functional impairment and greater risk of gait speed decline after cofounder control.
(27)	1999	Oregon Brain Aging Study	Gait Speed using a 9.14 m course.	Total Brain Volume, Intracranial Volume, Ventricular Volume, Periventricular High Signal, Deep High Signal.	Yes	Ventricular volume and periventricular white matter high signal volume, but not total brain volume or deep white matter high signal, were correlated gait speed independent of age.
(28)	2003	ABC 1921 Study	Gait Speed using a 6 m course.	WML, Periventricular Lesions and Brain Stem Lesions.	No	Decreased gait speed correlated significative with an increased grade of brain stem lesions.
(29)	2000	NHLBI Twin Study	Gait Speed using a 2.43 m course (faster of two walks).	White Matter Hyperintensities, Total Cranial Brain Volume (TCB).	Yes	Above the median total brain volume but not white matter hyperintensity volume was associated with higher gait speed.

In total, a maximum of 7,026 independent individuals participated in the studies evaluated, with the median study size being of 191 participants, ranging from as low as *n* = 30 for the functional MRI study of gait speed (22) to *n* = 2,450 for the study with participants from the Cardiovascular Health Study (CHS) (26) published in 2005. None of the studies estimated a population effect by reweighting to the source or even general population (30).

### Findings by frailty and frailty components

#### Frailty

In total, 3 studies were identified that investigated the association of structural brain parameters with frailty using MRI (13, 15, 31). Two of these, Avila-Funes et al. and Newman et al., analyzed directly the frailty phenotype originally proposed by Linda Fried (7), and Jung et al. reported in a letter the association between white matter abnormalities and a Frailty Index conceptualized as a combination of basic and instrumental activities of daily living, physical performance, cognitive function and serum albumin level. This index showed a significant correlation (Spearman′s = 0.49, *p* < 0.001) with Fried Frailty (14). All studies conclude that a higher burden of White Matter Lesions (WML) volume was associated with the prevalence of frailty. In addition, the original study from Newman et al. in participants from the above-mentioned CHS found evidence for a higher number of infarct lesions and increased ventricular size in frail participants but no association with sulcus size. Furthermore, Avile-Funes et al. found that white matter integrity assessed using diffusion tensor imaging was less preserved in frail participants from the AMIage study (13). This study investigated the relationship between fractional anisotropy (FA, lower in frail vs. non-frail participants), axial diffusivity (AD, higher), radial diffusivity (RD, higher), and mean diffusivity (MD, higher) across white matter tracts including the corpus callosum, anterior limb of internal capsule, external capsule, and posterior thalamic radiations.

All these studies adjusted for major confounders such as age, gender, and major age-associated diseases and were nested in longitudinal cohort studies.

#### Grip strength

Hirsiger et al. evaluated the association between grip strength and structural/functional connectivity in the cingulum during resting state as obtained from DTI and fMRI respectively in 165, cognitively normal older participants (mean age 70.15) from the longitudinal healthy aging brain (LHAB) project of the University of Zurich, Switzerland (17). They found that an increase in FA in the cingulum bundle was positively associated with grip strength (*p* = 0.022) while an increase in mean diffusivity was negatively associated with grip strength (*p* = 0.018) in models adjusted for age, gender, education, and diastolic blood pressure. Resting state functional connectivity in the cingulum, more concretely the correlation between posterior cingulate cortex and medial prefrontal cortex BOLD signals, was not associated to grip strength (*p* = 0.270).

Seidler et al. evaluated the same study sample as Hirsiger et al. but looking at individual regions-of-interest (ROIs), using left primary motor cortex, left putamen and right cerebellum lobules V and VIII, all of them associated to hand motor performance (16). They found that resting state connectivity between the motor cortex, bilateral sensorimotor cortex and supplementary motor area was greater in participants with higher grip strength. They also found stronger connectivity between the putamen region, medial frontal cortex and precuneus, as well as between the cerebellar seeds, the frontal cortex and temporal regions associated with higher grip strength. In addition, cerebellar lobule V showed increased connectivity with lobules VIIIa and VIIIb with greater grip strength.

#### Gait speed

Twelve studies using data from 9 population studies investigated gait speed. 11 studies used structural MRI imaging for testing, among other aspects, the association between WML (*n* = 9) (18, 20, 21, 23, 24, 26–29) and gray matter (*n* = 2) (19, 25) with gait speed, while one study evaluated resting state networks using fMRI and their association with gait speed (22).

In each study relating gait speed to neuroimaging markers, gait speed was assessed differently. Nevertheless, all but one study used velocity in units of distance (m or cm) per second as outcome measure rather than time in seconds for walking a predefined distance.

Nine studies investigated the relationship between gait speed and WML. Generalized measures of WML were associated with slower gait in models adjusted for major confounders in eight out of nine studies. Only the NHLBI Twin Study, one of the earliest neuroimaging studies from the year 2000, did not report a significant effect, although the tendency was consistent with the other works. Some studies (23, 24, 27) also analyzed the effect of periventricular WML burden coming to the same conclusions. In these studies, deep WML volume was not associated with gait speed. In addition, Silbert et al. examined the effect of change in WML volume and concluded that the accumulation of WML was associated with increased gait slowing during follow-up. Rosario et al. additionally investigated the possibility of an interaction effect between WML and white matter integrity measured by FA in participants from The Health, Aging and Body Composition Study (HealthABC) and found that the association between WML volume and gait speed was not significant in high FA individuals.

Two studies by Rosano et al. (19, 20) in participants from the CHS and Age, Gene/Environment Susceptibility (AGES) study investigated the association between gray matter volume and gait speed. Using a ROIs approach of areas a priori known to be associated to mobility, they identified an association between small volumes in cerebellum and dorsolateral prefrontal regions (25) and prefrontal gray matter volume (19) with slower gait. In addition, brain atrophy—defined by an atrophy index computed as (intracranial volume–brain volume)/intracranial volume—but not cerebral infarcts were associated with reduced gait speed in the AGES study.

The only study investigating resting state connectivity via functional MRI in participants from the Central Control of Mobility in Aging (CCMA) study confirmed an association between well-established sensorimotor, visual, vestibular, and left fronto-parietal resting state networks and gait speed in older adults.

## Discussion

Neuroimaging techniques and in particular functional neuroimaging, a cornerstone for diagnosing and investigating cognitive decline and dementia in the elderly, are hardly used to identify biomarkers and risk factors associated to frailty. This is surprising given the close link between frailty and cognitive decline which has led to ≪cognitive frailty≫ becoming a major research topic (32–34). As of end of January 2018, only 3 studies directly assessed the association between frailty and neuroimaging markers identifying a relationship between an increased burden of white matter hyperintensities, lower fractional anisotropy and higher diffusivity with a higher prevalence of frailty. None of these studies evaluated connectivity or any other functional metric. Furthermore, among these studies, the different frailty components have received uneven attention with many more studies focusing in the relationship between neuroimaging markers and gait speed compared to handgrip strength. A higher burden of white matter hyperintensities has been associated to lower gait speed. Furthermore, lower fractional anisotropy and an increase in mean diffusivity were associated to low gait speed and grip strength.

More white matter hyperintensities and lower white matter structural integrity were found to be associated with an increased prevalence of frailty, lower grip strength and slower gait in all studies that investigated this neuroimaging risk factor and were considered for this review. These results support investigative efforts into the role of the central nervous system and vascular damage as possibly being implicated in the pathophysiology of frailty. Findings supporting these results highlight the association between structural changes and WML with physical fitness and activity (35). In fact, white matter hyperintensities, possibly the result of arteriosclerotic processes, are almost ubiquitous in the elderly (36) and their presence is facilitated by the exposure in mid-life to well-known risk factors such as smoking, hypertension, diabetes mellitus, and chronological age (37). They are also consistently associated with cognitive impairment (37). However, to be associated with global cognitive decline, the presence of other lesions is required and by themselves they cannot be used as an indicator of dementia (38). As such, it is problematic to infer the role of WML in the development of frailty from the knowledge available to date, particularly since most of the studies reviewed here and all that directly investigated the frailty phenotype are cross-sectional and the reported findings could be a result of reverse causation. Accumulating longitudinal evidence in the fields of stroke, dementia and mortality, supports the role of white matter hyperintensities as a risk factor for these endpoints. But the associations reported for frailty, whether causal or not, might not be sufficient to back the classification of WML as a risk factor useful in the diagnosis or prognosis of frailty. Whether or not WML can provide useful information in combination with other biomarkers from the brain or OMICs remains to be evaluated.

White matter microstructure has been associated to frailty and its defining phenotypes in this review. DTI has emerged as a technique allowing the study of white matter changes occurring at a microscopic level before its macroscopic manifestations are visible on a structural MRI (39). DTI seeks to evaluate the loss of white matter microstructure integrity by characterizing the degree of restriction to movement across different ellipsoid axis (AD, RD, MD) as well as the relative degree of anisotropy in a region of interest indicative of a preferential diffusion path. DTI's sensitivity to subtle abnormalities has encouraged its application to the study of the aging brain under both healthy and pathological conditions, yet only two of the studies considered in this review deal directly with the microstructural alterations—as extracted from the exploration of DTI parameters—regarding frailty condition (13) or frailty-related components (17). The first study informed of a greater loss of WM integrity (lower FA and higher diffusivity values) in frail participants. Local decreases in FA have been also observed in normal aging—involving frontal WM and anterior cingulum—while DTI abnormalities found in participants undergoing cognitive decline (MCI) or neurodegenerative disease (AD) are also significant in posterior regions signaling a loci of irregularities that could be related to an Alzheimer's disease type pathology [for a systematic review, see (40)]. One of the regions reported in Avila-Funes et al. to exhibit a lower FA in frail older adults is the anterior limb of the internal capsule. This region has been subjected to some discrepancy in the MCI and AD literature. Some authors do not find significant reductions in FA (41, 42) while others do (43). The later suggest that motor dysfunction is part of the incipient process of AD but as this is not often clinically supported is thought to represent an uncommon subgroup within AD patients (40) that could be related to those individuals manifesting both a cognitive decline and a frailty condition. The anterior limb of the internal capsule, pinpointed in the study of Avila-Funes et al., is involved in the connection of frontal regions with different brain regions. Interestingly, frontal structural disconnection has been linked to cognitive decline in older adults, which seems to support the link between frailty and cognition. In Hirsiger et al., reduced grip strength was associated to the loss of WM microstructural integrity in the cingulum, a region whose fibers have been reported to present a significant FA reduction in MCI and even more in AD (44).

Many of the studies covered in this work agreed on the finding that brain volume reductions — manifested as either ventricular volume increase (15, 26, 27) or a diffuse reduction in total brain volume (20, 29)—are associated to classical phenotypes of frailty. However, the specific cortical atrophy pattern associated to physical frailty is yet to be fully established as very little work has addressed this question. In this vein, two of the studies reported significant reductions in prefrontal volume linked to slower gait speed (19, 25), which could shed some light in this regard. Gray matter atrophy is a hallmark of dementia progression and is closely linked to cognitive dysfunction (45). Interestingly, Silbert et al. (23) failed to find any significant relationship between gait speed and hippocampal volume, which is one of the first structures showing volume reduction in Alzheimer′s Disease dementia (46). Nevertheless, it is important to bear in mind that the specific pattern of gray matter atrophy is highly dependent on the dementia cause. Interestingly, the comorbidity between physical frailty and cognitive deterioration leading to dementia observed in epidemiological studies, seems to be supported by the fact that frailty has been consistently linked to gray matter atrophy in the few neuroimaging studies available to date, which is solidly known to be also a major risk factor for dementia development (47).

Functional connectivity estimates the reciprocal interactions between distant brain regions as a function of the statistical dependence between their respective activity time courses. Synchronous activity has been reported to be consistently associated to cognitive (48) and even motor performance (49). However, although its influence in cognitive deterioration and dementia is receiving increasing attention, its role has been very scarcely studied in the context of frailty. From the reviewed literature only three works reported functional connectivity metrics. Hirsiger et al. (17) failed to find any statistical relationship between posterior cingulate cortex-medial prefrontal cortex connectivity and grip strength. This particular link represents one of the major features of the default mode network (DMN), which is associated to internal processing states and is a critically associated to dementia progression (50). However, the other two studies employing FC metrics (16, 22) included a larger set of regions in their analyses obtaining in both cases similar results, highlighting a significant hyposynchronization affecting particularly sensorimotor areas and prefrontal regions. Although sensorimotor network is not one of the key networks in dementia progression, fronto-parietal network disruption (as reported by Yuon et al.) has been extensively linked to cognitive deterioration, particularly in attention and executive functions. This particular pattern of alterations could underlie the observed relationship between frailty and dementia risk. In general, functional neuroimaging techniques, such as MEG, have shown great utility in detecting the initial stages of dementia and its associations with amyloid-beta [for a review see (51)], which could be an important factor in explaining the link between frailty and dementia.

## Limitations

This scoping review has important limitations. First, the restrictions to the Fried phenotype and the non-diseased, elderly (65+ years) population, might have significantly reduced the study base. However as frailty phenotype is both more prevalent and potentially impactful in the older population we focused our review in that specific segment of the population. Nonetheless, to the best of our knowledge, this is the first review addressing the use of neuroimaging markers in frailty research, thus making it important to focus on research that approaches frailty from a broad perspective and in the non-diseased population to avoid coming to conclusions biased by results from specific diseased groups. Furthermore, although frailty definitions different from the Fried phenotype model exist (6, 52), it is still the most commonly employed. Second, as most of the studies reviewed are cross-sectional, reverse causality cannot be excluded, and the results reported here should be considered as mere statistical associations. Third, as in all observational research, residual confounding that artificially creates a statistical association between neuroimaging markers and frailty due to a common, unknown factor cannot be excluded. Fourth, as this review was restricted to the general, non-diseased population, we did not include different studies pinpointing a link between frailty and beta-amyloid accumulation in AD-related regions in at-risk population (53, 54). However, these studies could also be considered a very promising direction for future research into the relationship between dementia or cognitive dysfunction and frailty.

In conclusion, current literature supports the association between increased burden of white matter lesions, lower fractional anisotropy, and higher diffusivity with frailty and an overall worse performance in the different frailty components (i.e., gait speed and handgrip strength). However, the overall study base contributing to these findings is very small, mostly cross-sectional and does not allow for generalizations. Representative, longitudinal neuroimaging studies, structural and functional, investigating frailty and the subgroup of people that exhibit frailty and cognitive decline as comorbidity are urgently needed to identify processes that are specific to frailty or common to both frailty and cognitive decline and dementia to facilitate the differential diagnosis in the clinical setting.

## Author contributions

SW had the original idea of the manuscript, participated in the literature search and selection, and authored the initial draft of the manuscript. DL-S, IS-M, RB, NP participated in the literature review, the abstract screening, and the results extraction, and authored sections of the manuscript. FM, LR-M provided important intellectual input to the manuscript. All authors read, contributed, and approved the final version of the manuscript.

### Conflict of interest statement

The authors declare that the research was conducted in the absence of any commercial or financial relationships that could be construed as a potential conflict of interest.
